# Comparison the Effect of Bromelain Enzyme, Phosphoric Acid, and Polyacrylic Acid Treatment on Microleakage of Composite and Glass Ionomer Restorations

**DOI:** 10.30476/DENTJODS.2021.88737.1355

**Published:** 2022-06

**Authors:** Farahnaz Sharafeddin, Paniz Moraveji

**Affiliations:** 1 Dept. of Operative Dentistry, Biomaterials Research Center, School of Dentistry, Shiraz University of Medical Sciences, Shiraz, Iran; 2 Undergraduate Student, Dept. of Operative Dentistry, School of Dentistry, Shiraz University of Medical Sciences, Shiraz, Iran

**Keywords:** Bromelain, Glass ionomer, Polyacrylic acid, Phosphoric acid, Composite resin

## Abstract

**Statement of the Problem:**

Resin modified glass-ionomer cement (RMGIC) shows low microleakage values. Bromelain enzyme is a deproteinizing agent with an anti-inflammatory effect in human body.Efective cavity treatment is an important factor in reduction of microleakage.

**Purpose:**

The aim of this study was to determine the effectiveness of the deproteinizing aspect of 10% bromelain enzyme on the microleakage of RMGIC and composite restorations.

**Materials and Method:**

In this experimental study, 40 non-carious extracted human molar teeth were categorized in eight experimental groups (n=5). Standard class V cavities were prepared on the buccal and lingual surfaces of the teeth (n=10). The specimen were classified as Group 1, in which 20% polyacrylic acid (PAA) was applied on the teeth then treated with 10% bromelain enzyme; Group 2: 10% bromelain enzyme was applied; Group 3: 10% bromelain enzyme was applied and then treated with polyacrylic acid; Group 4: 20% polyacrylic acid was applied. Groups1 to 4 were restored with RMGIC (Fuji II LC, GC, Japan). Group 5: etched by 37% phosphoric acid and then treated by 10% bromelain; Group 6: 10% bromelain enzyme was applied without etching; Group 7: teeth were deproteinized with 10% bromelain enzyme and then etched with 37% phosphoric acid; and Group 8: cavities were etched with 37% phosphoric acid. In the groups 5 to 8, Adper single bond (3M, ESPE, USA) and filled with composite resin Z350 (3M, ESPE, USA). After thermocycling, the teeth were sectioned. Microleakage scores were measured using stereomicroscope (40×). Kruskal-Wallis and Mann-Whitney tests were used for data analysis. (*p*< 0.05)

**Results:**

Statistical analysis did not show any significant difference in occlusal and gingival margin microleakage in glass ionomer groups (1-4) (occlusal *p*= 0.218, gingival *p*= 0.192). Kruskal-Wallis revealed significant difference in occlusal and gingival margin microleakage of Groups 5 to 8 (occlusal *p*= 0.006 and gingival *p*= 0.00). Group 5 demonstrated the lowest occlusal microleakage (occlusal mean=0.00).

**Conclusion:**

Applying bromelain or polyacrylic acid did not affect the microleakage of glass ionomer filling. Due to the antinflamatory effects of bromelain, we suggest using it instead of PAA. Pretreatment of 10% bromelain enzyme after phosphoric acid significantly decreased microleakage in the occlusal and gingival margin of composite filling.

## Introduction

Request for aesthetic restoration has led to the introduction of different tooth-colored restorative materials such as glass ionomer cements (GICs) and composite resins. GICs adhere to the enamel and dentin with fluoride release, and have low cytotoxicity and microleakage [ 1 - 3 ]; however, they have low toughness and strength [ 4 - 7 ]. Composite resin has an important role in esthetic dentistry, but polymerization and shrinkage cause a volumetric reduction of resin. Rapid polymerization and volume loss may lead to gap formation and debonding that cause breakdown in the margine of the restoration. Conditioning is required for achieving an effective adhesion between the tooth structure and the GICs. Polyacrylic acid (PAA) is a traditional conditioner in GICs restorations. Conditioning of the dentin causes the chemical reaction between GIC and hydroxyapatite crystals and can demineralize partially the dentine surface [ 8 ].

The microleakage causes the permeability of chemical ions and bacteria and leads to postoperative sensitivity, recurrent caries, pulpal pathology, and failure of the restoration. Many strategies have been used to increase restoration bond strength, reduce the microleakage, and future failures such as degradation of collagen fiber [ 9 - 12 ]. One of these materials is bromelain, which is a proteolytic enzyme extracted from pineapple. It has many properties like reducing tissue inflammation, pain, and edema [ 13 ]. Investigation revealed that application of bromelain enzyme leads to the removal of collagen network and a significant decrease in the global leakage of the adhesive system [ 14 ]. Moreover, it has been reported that removal of unsupported dentin collagen fibers with bromelain enzyme after acid etching results acceptable bond strength [ 15 ].

As there were a few investigations that report the effect of bromelain on microleakage of composite and RMGIC restorations, this study was conducted to determine the exact function of bromelain enzyme and PAA and phosphoric acid treatment of cavity in composite and RMGIC restorations.

## Materials and Method

A total of 40 human intact extracted third molars were selected in this experimental study. They were stored in 0.1% thymol solution for 48h. They were mounted 4 mm apically to CEJ in cylindrical acrylic resin with 6 cm height and 3 cm diameter. 

Diamond fissure bur (330, SS White, USA) was used for every 5 preparations in a high-speed handpiece with water and air spray. Class V cavities (3mm in width, 5mm in length and 2mm in depth) [ 17 ] on the buccal and lingual surfaces of each tooth was prepared, where the gingival margin of cavities was 1 mm below the CEJ. The teeth were randomly placed into 8 groups of 10 cavities and then filled as follows.

In the Group 1, 20% PAA (GC, Tokyo, Japan) was applied for 20 s by a microbrush, rinsed for 20 s with water, and dried gently. Bromelain powder (Salamat Parmoon Amin manufacture, Iran) was dissolved in distilled water to obtain 10% bromelain enzyme. The solution was applied on the cavity surfaces by a microbrush for 60 s, rinsed for 20s and dried [ 12 ].

In the Group 2, the bromelain enzyme was applied directly into the cavities for 60 s, rinsed for 20 s, and dried. In the Group 3, the teeth were treated by bromelain solution for 60 s then rinsed for 20 s. PAA 20% was applied by a microbrush for 20 s, rinsed for 20 s, and dried. In the group 4, PAA 20% was applied, rinsed for 20 s, and dried. All specimens in Groups 1 to 4 were restored with RMGIC (Fuji II LC, GC, Japan). RMGIC was prepared according to the manufacturer’s instruction. The liquid and powder was mixed with the ratio of 3.2:1 by weight for 25 s using clean slab and a plastic spatula. The cavity was filled then cured with LED light-curing unit (Light curing unit, Demi plus, Kerr, Switzerland) with the light intensity of 1200 Mw/cm^2^ for 20 s and less than 1 mm distance from the restoration surface. The varnish was applied to the surface of all four groups (n=20). In the group 5, the teeth were etched for 20 s with 37% phosphoric acid gel (Den fill Etchant-37, LTM, Korea), rinsed for 20 s with water, and air-dried gently. The bromelain enzyme was applied into the cavities for 60 s and then rinsed for 20 s. The dentin was coated by Adper single bond (3M, ESPE, USA) using microbrush and cured for 20 s. In the group 6, the bromelain enzyme was applied for 60 s and then rinsed for 20 s and air-dried. A coat of Adper single bond was applied on the dentin surface and cured for 20 s. In the group 7, the cavities were treated by bromelain enzyme for 60 s, rinsed for 20 s, and then 37% phosphoric acid gel was applied for 20 s on all part of cavity according to manufacture recommendation [ 10 ]. The teeth were rinsed and gently air dried and Adper single bond was applied and cured. 

In the group 8, 37% phosphoric acid gel was applied for 20 s, rinsed for 20 s, and gently dried. Then Adper single bond was applied and cured. All specimens in groups 5 to 8 were filled by composite resin in two layers, (Z350; 3M, ESPE, USA) and cured for 40 s (perpendicular to the cavity, less than 1 mm distance). All groups were stored in deionized water for 7 days at room temperature (24℃). Then all restorations were finished with finishing burs and polished using course to fine polishing disks (Shofu, Tokyo, Japan). All specimens were thermocycled for 1000 cycles at 5±2℃/50±2℃ with a dwell time of 30 s using thermocycling machine (TC-300, Vafaie Industrial, Iran) [ 16 - 19 ] and then were stored in deionized water. In exception of the fillings and 1mm surrounded area, the teeth surfaces were coated by two layers of nail varnish. Then, the samples were stored in 2% basic fuchsine solution (Merck, German) for 24h at room temperature. After removal of the specimens, the superficial dye was washed with running water.

**Figure 1:**
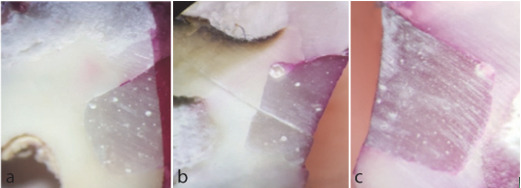
Microleakage scoring (×40): **a:** 0 = no dye penetration; **b:** 1= dye penetration up the one-half of the occlusal wall; 2= dye penetration extending beyond one- half of the distance but not reaching axial wall; and **c:** 3= axial wall dye penetration extending

Initially, the specimens were cut horizontally 2 mm below the gingival margin of the restoration and then longitudinally in a buccolingual direction from the mid part of each the restorations. Using a diamond disk (Microdont, Brazil) in a nonstop cutting machine (Demco E96, CMP Industries, USA) under a water spray. In a double-blind study, two undergraduate students evaluated the sectioned specimens under a stereomicroscope (Estscope Bs-3060, Best Scope, China) at 40 x. The extent of dye penetration at both gingival and occlusal margins were classified according to microleakage scores as (0) no dye penetration; (1) dye penetration between the restoration and the axial wall lesser or equal 1/2 of the distance; (2) dye penetration extended half of the distance but not reached the axial wall; and (3) dye penetration reached the axial wall [ 18 ] (Figure 1). 

All collected data were analyzed using IBM SPSS (Chicago, IL, USA) v. 22.0 (IBM Inc.). Kruskal-Wallis and Mann-Whitney tests were performed in order to compare the microleakage values between the groups (*p*< 0.05).

## Results

**Figure 2:**
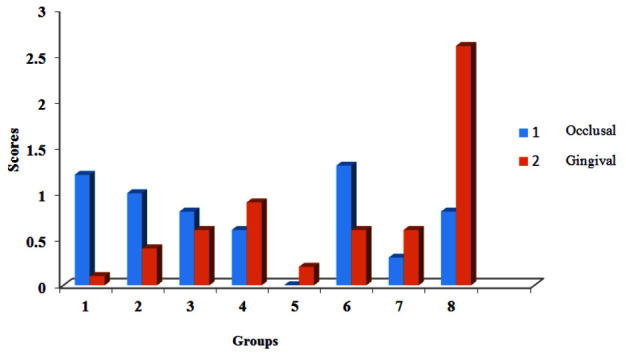
Mean of microleakage scores

The means and standard deviations (SD) of microleakage scores are illustrated in Tables 1 [Table T2] [Table T3] - 4 and Figure 2. 

**Table 1:** The Mean ±SD of occlusal and gingival margin microleakage scores of glass ionomer groups and *p* value of Kruskal Wallis test

Materials	Mean ±SD	
	Occlusal	Gingival
Poly acrylic acid + bromelain	1.20 ± 1.033	0.10±0.316
Bromelain	1.00 ±1.155	0.40±0.966
Bromelain+ Poly acrylic acid	0.80 ± 0.632	0.60±0.843
Poly acrylic acid	0.60 ±1.265	0.90±1.197
*p* Value	0.218	0.192

**Table 2 T2:** Mean ±SD occlusal and gingival margin microleakage scores of composite groups and *p* Value of Kruskal Wallis test

Materials	Mean ±SD	
	Occlusal	Gingival
Phosphoric acid+ bromelain	0.00±0.0000	0.20±0.422
Bromelain	1.30±1.337	0.60±1.075
Bromelain+ Phosphoric acid	0.30±0.483	0.60±0.843
Phosphoric acid	0.80±0.632	2.60±0.516
*p* Value	0.006	0.00

**Table 3 T3:** *p* Value of Mann-Whitney test in microleakage of occlusal and gingival margin of resin modified glass-ionomer cement in comparison with composite

Composite	Glass ionomer	Occlusal	Gingival
Phosphoric+Bromelain	Poly acrylic acid +Bromelain	0.001	0.542
Bromelain	Bromelain	0.690	0.619
Bromelain+Phosphoric	Bromelain+ Poly acrylic acid	0.067	1.000
Phosphoric	Poly acrylic acid	0.130	0.04

**Table 4:** Dunn test Pairwise *p* value comparison in composite groups in occlusal and gingival margin

Composite	Composite	Occlusal	Gingival
Phosphoric+ Bromelain	Bromelain	0.015	1.000
Phosphoric+ Bromelain	Bromelain+ Phosphoric	1.000	1.000
Phosphoric+ Bromelain	Phosphoric	0.028	0.000
Bromelain + Phosphoric	Bromelain	0.358	1.000
Bromelain + Phosphoric	Phosphoric	0.564	0.003

According to the results of Kruskal-Wallis test, there was no statistically significant difference in the occlusal (*p*= 0.218) and gingival (*p*= 0.192) margin microleakage scores of RMGIC groups. In composite groups, this test demonstrated a significant difference in occlusal (*p*= 0.006) and gingival (*p*= 0.00) margin microleakage. Mann-Whitney test showed a significant difference between group 1 and group 5 occlusal margin microleakage (*p*= 0.001). In the gingival margin, this test also revealed a significant difference between PAA (group 4) and phosphoric acid (group 8) (*p*=.04).

In Dunn pairwise test to pairwise comparison of occlusal margin microleakage in composite groups, the pairwise p value demonstrated a significant difference between groups 5 and 6 (*p*= 0.015), as well as groups 5 and 8 (*p*= 0.028). In gingival margin, this test showed a significant difference between groups 5 and 8 (*p*= 0.000), groups 6 and 8 (*p*= 0.002) and groups 7 and 8 (*p*= 0.003).

Means of Kruskal-Wallis test in occlusal margin showed that group 5 (Phosphoric acid+ Bromelain) had the lowest score (mean=0.00) compared to composite and RMGIC.

## Discussion

Treatment of dentine surface with phosphoric acid leads to dissolving mineral component of the smear layer and remaining amorphous protein layer, which decreases the rate of adhesive resin penetration and forms a weak hybrid layer that subsequently reduces the composite bond strength [ 10 ]. Bromelain enzyme can eliminate organic component and collagen from the surface of the dentin and increase resin penetration into dentin structure, improve the hybrid layer, and decrease microleakage [ 20 ].

In our study, pretreatment of 10% bromelain enzyme after phosphoric acid significantly decreased microleakage in the occlusal and gingival margin of composite filling, which is in accordance with the results of previous studies. In contrast, we noticed that,etching the cavity after bromelain enzym aplication does not have much impact on microleakage reduction, especially in gingival margin. 

It has been reported that using bromelain enzyme on etched dentin surface significantly decreased marginal microleakage. The ability of bromelain to remove collagen network of etched dentin surface is important [ 14 , 21 ]. In similar results, bromelain enzyme increases the permeability of dentin surface by depletion of collagen fibril from the acid-etched surface and causes widening of dentinal tubules in the outer surface of dentine. It also increases the dentine surface energy and enhances penetration and infiltration of adhesive monomers into dentin [ 15 ]. Thus, this finding is in accordance with our results, may be because of the arranged application of phosphoric acid and bromelain after each other. In this regard, in another group, we applied bromelain before acid and did not see a significant decrease in occlusal and gingival margin microleakage. As bromelain is an anti-inflammatory agent and can affect stem cells, it seems that the use of this material may reduce the hazard of composite material on dental pulp [ 22 ]. Deep tooth colored restorations may influence the dental pulp. In deep carious teeth with mild pulpits, the use of anti-inflammatory agent instead of acids may reduce the pulpitis. Therefore, in the current study, bromelain was applied for one minute and then was washed.

In one study [ 16 ], the effect of phosphoric acid-etched dentin surface with 5% bromelain enzyme and Nd:YAG laser prior to the use of etch and rinse adhesive systems on microleakage margins of class V composite restorations has been reported. They showed that gingival margins microleakages were significantly higher than the occlusal margins. Thus, they concluded that application of proteolytic agents on acid-etched dentin surface prior to the application of adhesive has no significant effect on marginal microleakage of class V composite restorations [ 16 ]. This finding is opposed to our results. The difference may be related to the priority and arrangement of our study, in which bromelain was applied after phosphoric acid, and also the bromelain dilution amount, laser application, and the type of composite used. Moreover, in our study, application of bromelain after acid etch in the occlusal margin showed better results; however, in both occlusal and gingival margin, it caused a significant reduction in microleakage [ 23 ].

In our study, the statistical analysis showed that etching dentin, with 37% phosphoric acid alone, did not decrease the microleakage significantly.

In one study [ 24 ], researchers examined the etching effects of phosphoric acid versus a combination of phosphoric and hydrofluoric acid by evaluation of microleakage in a composite restoration bonded with different dentin adhesive systems. This study showed that a combination of phosphoric and hydrofluoric acid led to significant reduction in dye penetration compared to phosphoric acid conditioning per se [ 24 ]. This result suggests that phosphoric acid would not be able to decrease microleakage when used alone. In our study, when phosphoric acid was applied alone, most microleakages were observed in the gingival margin and a considerable leakage was seen in the occlusal margin. It seems the combination of phosphoric acid with another material such as hydrofluoric acid or bromelain enzyme can increase the capability of phosphoric acid to reduce microleakage.

The use of conditioning before placing the GICs significantly increases the ionic bond to dentin. PAA is a very weak acid that does not significantly demineralize dentinal tissue nor increase the likelihood of postoperative sensitivity [ 25 ]. It has been reported that pretreatment with a weak PAA conditioner has the ability to remove the smear layer and partially demineralize the dentin [ 26 ]. In our study, we applied PAA before GIC and it could reduce the microleakage to some extent. However, the difference was not significantly different when bromelain was used alone and even in the gingival margin, application of bromelain enzyme alone showed better results than occlusal margin concerning the microleakages.

The effect of two traditional PAA conditioners and 2% chlorhexidine (CHX) digluconate on cavosurface microleakage of glass ionomer restorations has been evaluated [ 27 ]. It was reported that 2% CHX digluconate was as efficient as the other conditioners. No statistically significant differences were found among the three types of conditioners. Dye penetration was significantly greater into gingival than into occlusal among all three conditioners in both groups. In addition, 2% CHX digluconate, with its known added advantages, can be used as a pretreatment conditioner in GIC restorations [ 27 ]. This can be in accordance with the results of our study that showed although PAA in occlusal and gingival margin could reduce microleakage to some extend, it is better to use a substance that is safer and non-allergic and has anti-inflammatory effects compared to an acid that causes sensitivity. The present study revealed that in GIC groups, there was no significant difference in occlusal and gingival margin microleakage values among four groups. Accordingly, in the occlusal and gingival margin, the use of bromelin alone or in combination with PAA, did not decrease the microleakage; so, it can be replaced with each other. CHX acts as matrix metalloproteinases inhibitor and by preventing dentin collagen degradation can preserve the resin- dentin bond strength up to 6 months [ 28 ]. CHX mouthwash has a toxic effect on fibroblast cells but there is no evidence of cytotoxicity of bromelain in the literature [ 29 ]. In their study, CHX was kept in the cavity but in our study, bromelain was rinsed. Therefore, bromelain enzyme application for more than 60s in the cavity may show better results. The results of their study are similar to ours but the mechanism of action differs prominently between bromelain and CHX.

In one research [ 30 ], the microleakage of RMGIC and conventional GICs liners in Class V composite and GIC restorations has been measured by measuring the amount of microleakage at the gingival margins and reported no differences among the groups using the RMGIC. Removal of the smear layer using 10% PAA acid did not influence microleakage in restorations with RMGIC liners [ 30 ]. Although the method differs between these studies, such a result is similar to the outcomes of the present study, in which PAA did reduce the microleakage in both occlusal and GIC restoration groups. Hence, as bromelain and PAA showed almost similar microleakage values, it is better to use bromelain instead of PAA to prevent sensitivity and irritation of pulp. Finally, it is important to know that PAA and other cavity conditioners may cause tooth sensitivity [ 31 ]. Accordingly, applying safe and nontoxic materials in order to decrease inflammation is of great necessity. Bromelain is cheap, easy to access, harmless, and easy to use. Hence,the use of this material is recommended for reducing the microleakage of composite fillings.

This is the first study that compared the microleakage when phosphoric acid, PAA and bromelain were employed with composite and glass ionomer fillings. In our study, application of bromelain after acids in the occlusal and gingival margin of composite filling showed a statistically significant difference in marginal microleakage compared with glass ionomer filling, which may be due to the effect of other influential factors such as percentage and duration of use of bromelain. In this study, 10% bromelain was applied for 60 s in order to simulate short clinical application and had the lowest possible toxicity. Application of bromelain enzyme on conditioned dentin significantly decreases the values of the global leakage score and gives the lowest values of global leakage scores. This result can be explained by the ability of bromelain enzyme to remove the collagen network from acid-etched dentin efficiently. As a result, an increase may occur and the diffusion potential of the monomer to the intact dentin and the microleakage would be minimized. Furthermore, removing the collagen network from acid-etched dentin substrate will make the chemical composition of dentin more similar to that of enamel by minimizing the organic component of dentin substrate and this will lead to the changing of the hydrophilic properties of the dentin [ 11 ]. Future *in vivo* studies should be done because it differs from in vitro considering the absence of saliva, different microorganism colonization, pH, thermal condition, nutrition, and variation of tooth morphology. In addition, we suggest conducting similar studies using different types of bonding systems with variable pH, self-etchants, and different concentration of bromelain.

## Conclusion

Considering the limitations of this research, using bromelain alone in RMGIC fillings showed almost a similar microleakage in occlusal and gingival margin of PAA group. The most important result of this research is that bromelain can be used as an organic and harmless material with less damaging effect on tissues instead of chemical hazardous acid in operative dentistry. In this study, from composite filling, we concluded that the application of bromelain after phosphoric acid reduces microleakage in occlusal and gingival margins. Hence, it is recommended to use bromelain in composite fillings.

## Acknowledgement

This project (No.76-15402) was carried out by financial support from the Deputy Dean of Research at Shiraz University of Medical Sciences. The authors would like to thank Dr. Mehrdad Vosoughi for statistical analysis of the data.

##  Conflict of Interest:

There are no conflicts of interest.
